# Letter to the editor: Failing an automated comprehension test while being able to report on needs: eyetracking in critically ill intubated patients should not be underestimated

**DOI:** 10.1186/s13054-022-04280-x

**Published:** 2022-12-22

**Authors:** Christian Waydhas, Robert Gaschler, Christopher Ull, Christina Weckwerth, Oliver Cruciger, Uwe Hamsen

**Affiliations:** 1 Department of Trauma Surgery, University Hospital Essen, Hufelandstraße 55, 45147 Essen, Germany; 2 Department of General and Trauma Surgery, BG University Hospital Bergmannsheil, Bürkle-de-la-Camp-Platz 1, 44789 Bochum, Germany; 3 Department of Psychology, FernUniversität Hagen, Universitätsstraße 33, 58084 Hagen, Germany

Dear editors

With great interest, we have read the article “Assessing oral comprehension with an eye tracking based innovative device in critically ill patients and healthy volunteers: a cohort study” published by Bodet-Contentin and colleagues in *Critical Care*.

They studied the ability of healthy volunteers and critically ill patients to answer the questions of the Montreal Toulouse Test to study their oral comprehension by using an eye-tracking device. The test questions were read out loud by a recorded voice, and the test was performed automated without the active involvement of a human being. An answer was classified as “right” when the subject fixated the panel with the described picture for at least 3 s within a time window of 6 s. The authors reported a median rate of correct answers of 93% in healthy volunteers and of 38% critically ill patients. As a take-home message, they conclude that “implementing an oral comprehension test using an innovative eye-tracking-based interface seems feasible in critically ill intubated patients.”

Critical readers may come to another conclusion. They might take home that most critically ill patients who are thought to be able to communicate judiciously (calm and awake as evaluated by the Richmond Agitation and Sedation Scale (RASS) with proper hearing and vision) are not able to do so by using eye-tracking technology. Performance of critically ill patients on the test apparently was very poor. Of the 15 test items used (compare: video provided online as File 2), nine contained four panels (i.e., one target panel and three distractor panels, guessing baseline = 25%). The other 6 contained two panels (guessing baseline = 50%). The average guessing rate across the 15 items thus was 35%. Hence, the performance of critically ill patients was very close to the performance expected under guessing. For critically ill patients older than 60, the authors report an even lower performance (27% median correct rate). One might argue that—instead of exclusively arguing based on guessing rates—one should also consider the time demands and constraints (fixating the correct panel for 3 out of 6 s). Yet, in this case one would still conclude that critically ill patients apparently perform very poorly on the test the way it had been administered. Given that their performance was close to chance baseline, one might even doubt that eyetracking-based assessment is feasible at all in this population.

However, we feel that some technical concerns may have precluded more favorable results, as several other investigators have observed [2–4].

- Our major concern is that the requirement of gazing at least three seconds at a panel on a monitor in the study by Bodet-Contentin et al. is not possible for many ICU patients. In the study from Duffy et al. [2], a gaze fixation of 0.4 s was used. In another study, it has been observed and reported by patients that even a gaze fixation time of 1 s was too strenuous for many patients [5] and a gaze fixation time of 0.6 s was recommended. This is one reason: We believe that the results from Bodet-Contentin substantially underestimate the ability of their patients to thoughtfully communicate by using the eye-tracking technology.
- Another reason lies in the observation that intubated critically ill patients can consistently report on their appraisals of their situation via eye fixations on response panels [6]. For instance, 90% indicate to feel trapped (while other items show lower approval rates). This underlines that patients understand what they are asked and are able to indicate their answer via fixation position. The questions posed in the Montreal–Toulouse test do have little or no context to the extreme situation the patients are experiencing. Accounting for the high level of concentration required by the patients their motivation to give answers might be reduced. Beyond the example just mentioned, in several studies it has been shown that critically ill patients are quite able to give differentiated answers questions concerning their actual situation, requirements, and projections on their future [3–6].
- Thirdly, although automation of tasks will allow the medical personal on an ICU to focus on other duties, in this particular setting, we feel that a personal interaction with a human being would be preferable. One major concern of awake critically ill patients is the lack of communication with nurses or physicians [7]. So, being interrogated by a machine (e.g., automation, recorded voice, lack of individualization) might further reduce patients’ motivation and ability to focus their gaze.

In conclusion, we congratulate the authors that they provided a study on assessment of oral comprehension after previous studies suggested that the eye-tracking technology is feasible in non-verbal critically ill patients. On the other hand, we believe that some of the unfavorable technical circumstances of the study did preclude a better performance of those non-verbal patient. Future studies have to show that the eye-tracking technology can be successfully implemented in the daily routine in an ICU and adds to the well-being of non-verbal patients.

## Data Availability

Not applicable.

## Abbreviations

ICU: Intensive Care Unit
RASS: Richmond Agitation and Sedation Scale
